# Incidence of Clinically Significant Neutropenia and Complications Related to Antibody-Drug Conjugates: A Real-World Study at the University of California

**DOI:** 10.3390/cancers18101563

**Published:** 2026-05-12

**Authors:** Tiffany Jan, Miranda Chen, Fan-Ying Chan, Nicole Kuderer, Alexandre Chan

**Affiliations:** 1 School of Pharmacy & Pharmaceutical Sciences, University of California, Irvine, CA 92697, USA; tpjan@hs.uci.edu (T.J.); xinyic39@hs.uci.edu (M.C.); fanyinc@uci.edu (F.-Y.C.); 2 UC Irvine Health, Orange, CA 92868, USA; 3 Division of Hematology, Department of Medicine, University of Washington Medical Center, Seattle, WA 98195, USA; kuderer@u.washington.edu

**Keywords:** antibody-drug conjugates, neutropenia, febrile neutropenia

## Abstract

**Background/Objectives**: Using real-world data, this study evaluates the use of ADCs over a decade across six University of California (UC) medical centers, focusing on the incidence of neutropenia-related adverse effects and associated clinical outcomes. **Methods**: A real-world retrospective study utilizing UC Health Data Warehouse records of patients receiving at least one dose of the ten most-used ADCs at one of the 6 UC hospitals between 2012 and 2024. The outcomes evaluated in this study were grade 3+ neutropenia and febrile neutropenia (FN) during any treatment cycle, and healthcare utilization outcomes including G-CSF prophylaxis, hospital and ICU admission, and mortality. Multivariate logistic regression was conducted to identify predictors for neutropenia-related events after the first dose of ADC. **Results**: Data from 3511 patients (mean ± SD age: 56.7 ± 17.6 years) were analyzed. The most commonly observed cancer types were breast cancer (43.4%), lymphoid, hematopoietic (41.8%), and urinary tract cancer (13.4%). The top three most commonly prescribed ADCs were fam-trastuzumab deruxtecan (22.6%), ado-trastuzumab emtansine (19.4%), and brentuximab vedotin (18.1%). Their respective all-cycle rates of FN were 5.4%, 1.2%, and 10.4%, while grade 3+ neutropenia rates were 16.4%, 5.1%, and 40.1%. Gemtuzumab ozogamicin and inotuzumab ozogamicin were associated with the highest rates of FN (both 18.1%), and inotuzumab ozogamicin was associated with the highest rates of hospital admissions (27.1%), ICU admissions (3.8%), and deaths (5.2%). Anemia and immunodeficiency disorders were identified as predictors for first-dose FN among patients receiving ADCs. **Conclusions**: Real-world data from this large multi-center cohort showed substantial variation in neutropenia-related events across ADCs. As ADC use continues to grow, these findings highlight the need for vigilant monitoring and proactive supportive care.

## Simple Summary

Antibody-drug conjugates are cancer treatments that deliver powerful chemotherapy directly to tumor cells, reducing harm to healthy tissue. While these drugs can be more effective and safer than traditional chemotherapy, they can still cause serious side effects, in particular low white blood cell counts, which increase infection risk and hospital visits. In this study, we analyzed real-world data from 3511 patients across six University of California hospitals to understand how often these blood-related complications occur with different antibody-drug conjugates. We found that the risk of serious infections and low white blood cells varied widely depending on the drug, and certain conditions, like anemia or weakened immunity, increased the risk. These findings can help doctors better monitor and manage side effects and provide insights for researchers developing safer and more effective targeted cancer therapies.

## 1. Introduction

Antibody-drug conjugates (ADCs) represent a class of targeted cancer therapies that combine the specificity of monoclonal antibodies (mAbs) with the potent cytotoxic effects of chemotherapy [1]. Designed to selectively target and destroy malignant cells while sparing healthy tissue, ADCs function by binding to tumor-specific antigens that are overexpressed on the surface of cancer cells. Currently, the FDA has approved usage of ADC for treatment of multiple malignancies, such as breast cancer, bladder cancers, and leukemia [2,3,4,5]. Clinically, ADCs offer several advantages over traditional chemotherapy. ADCs minimize systemic exposure and off-target toxicity, resulting in an improved therapeutic index [6]. This targeted approach allows for the administration of higher doses of cytotoxic drugs with greater safety and tolerability. Furthermore, ADCs have shown promise in overcoming multidrug resistance and are increasingly used in cases of advanced or refractory malignancies where conventional treatment options have failed [7,8].

Despite these advantages, ADCs are not without risk. Hematologic toxicities, particularly neutropenia and febrile neutropenia, are among the most common and clinically significant adverse events associated with ADC therapy [9,10]. A systematic review and meta-analysis by Zhu et al. analyzed clinical studies conducted between 2000 and 2022, encompassing over 17,000 patients treated with more than 28 different ADCs. The analysis found that neutropenia occurred in 43.7 percent of cases, while febrile neutropenia was reported in 27.5 percent of patients, both of which substantially increase the risk of infection and hospitalization [10].

Currently, few studies focus on the burden of hematological toxicities of ADCs as a whole [11,12]. Most pharmacovigilance studies provide context on specific ADC drug safety. For example, real-world analysis of the FDA Adverse Event Reporting System (FAERS) database from 2020 to 2022 identified prominent neutropenia-related safety signals with sacituzumab govitecan, particularly among geriatric patients [13]. Similarly, a multicenter retrospective study by the Hellenic Cooperative Oncology Group evaluated trastuzumab deruxtecan and sacituzumab govitecan in 312 pretreated patients with metastatic breast cancer, reporting neutropenia in 12.6% of patients receiving sacituzumab govitecan [14]. These studies highlight the importance of studying the real-world relevance of hematologic adverse events associated with ADC therapy.

The purpose of this study is to evaluate the incidence of clinically significant neutropenia and related complications in patients receiving ADC therapy within a real-world population at six University of California hospitals. This analysis aims to better characterize the hematologic safety profile of ADCs and inform future clinical management strategies.

## 2. Materials and Methods

### 2.1. Study Design

This study is a multi-center, observational, and retrospective analysis conducted using data extracted from the University of California (UC) Health Data Warehouse. The analysis includes patient records from six UC academic medical centers and their affiliated hospitals, all of which offer specialized oncology services and house NCI-designated Comprehensive Cancer Centers: UC San Francisco, UC Davis, UC Los Angeles, UC Irvine, UC San Diego, and UC Riverside-affiliated facilities [15].

The UC Health Data Warehouse (UCDWH) is a secure, centralized data platform that collects and stores clinical information from across the UC Health System, which includes six academic medical centers, 10 hospitals, and 18 health professional schools. It contains records of over 8.7 million patients seen since 2012, including more than 1.3 billion medication orders, and 5.2 billion vital signs and test results [15].

The study protocol was reviewed and deemed exempt from informed consent by the Institutional Review Board (IRB).

### 2.2. Inclusion/Exclusion Criteria

The study analyzed records from January 2012 to July 2024, focusing on patients treated with at least one dose of any of the following ten FDA-approved antibody-drug conjugates (ADCs): fam-trastuzumab deruxtecan, ado-trastuzumab emtansine, brentuximab vedotin, sacituzumab govitecan, enfortumab vedotin, gemtuzumab ozogamicin, inotuzumab ozogamicin, polatuzumab vedotin, belantamab mafodotin, and tisotumab vedotin. In this study, patients with missing medical record data were excluded.

### 2.3. Definitions

Given the retrospective nature of this study and its reliance on large administrative databases, clinical outcomes were primarily identified using a mix of diagnosis codes and laboratory results extracted from electronic medical record data. In this study, febrile neutropenia (FN) was defined using one of the following methods: (1) neutropenia (ICD-9 code 288.0) and fever (ICD-9 code 780.6); (2) absolutely neutrophil count (ANC) < 1000/mcL in the presence of fever (ICD-9 code 780.6) and (3) neutropenia (ICD-9 code 288.0) or ANC < 1000/mcL in the presence of a documented infection. We adopted this definition from two similar real-world studies that utilized a large claim database to evaluate neutropenia-related outcomes [16,17].

Grade 3 or higher neutropenia was defined in accordance with the Common Terminology Criteria for Adverse Events (CTCAE) version 5 as an absolute neutrophil count (ANC) of less than 1000 cells/mm^3^ within 28 days following the administration of the first ADC dose [18]. Hospital and intensive care unit (ICU) admissions related to neutropenia were identified using relevant diagnosis codes for neutropenia, fever, or infection [19]. Deaths were defined as those occurring either during the hospital admission or within two days following discharge, in order to capture early mortality potentially associated with neutropenic complications. Liver and renal dysfunction were defined using CTCAE laboratory grading based on the upper limit of normal from the reference range of each laboratory record [18].

Primary G-CSF prophylaxis was defined as administration occurring within 1–3 days following any ADC dose [20].

### 2.4. Outcomes

The primary outcomes evaluated in this study were the incidence of febrile neutropenia and grade 3 or higher neutropenia during any treatment cycle. Secondary objectives included determining the incidence of healthcare resource utilizations including G-CSF primary prophylaxis use, frequency and outcomes of hospitalizations, ICU admissions, and mortality. Additionally, characteristics associated with clinically significant neutropenic events at the first cycle of ADCs were identified. Together, these endpoints provide insight into the clinical impact of neutropenic adverse events and the role of supportive therapies in patients treated with ADCs.

### 2.5. Statistical Analysis

Descriptive statistics were employed to summarize both patient characteristics and clinical outcomes. For categorical variables, results were reported as percentages to provide a clear overview of their incidence. Continuous variables, such as patient age, were summarized using both the mean and median, along with standard deviation, depending on the underlying distribution of the data.

Chi-square tests and Fisher’s exact tests were conducted for univariate analysis to evaluate the association of patient characteristics with neutropenia-related outcomes. These characteristics include basic demographics, ADC type, malignancy type, comorbidities, pre-existing conditions, G-CSF use. Variables with *p* < 0.05 or clinical relevance in the univariate analysis were included in multivariate logistic regression models. Effect sizes were reported as adjusted odd ratios (OR) and 95% confidence intervals were presented for each of the independent predictors. Stratified analysis based on the various tumor status (solid vs. hematological malignancies) was also conducted. R version 4.4.2, which is embedded in the UCDWH’s databricks, was utilized for the analysis.

## 3. Results

Patients included in this descriptive analysis were identified from the UC Health Data Warehouse and had received at least one dose of the top 10 most commonly used ADCs. Application of the inclusion criteria resulted in a final cohort of 3511 patients. Depending on the ADC, the average time between cancer diagnosis and ADC initiation range from 290 to 1655 days (Table S1).

### 3.1. Baseline Characteristics

The baseline characteristics of the study cohort are summarized in Table 1. The mean age of patients was 57 years. The majority of patients were female (65.7%), and over half weighed ≥60 kg. In terms of race, most patients identified as White (52.7%). A significant portion of the cohort (59.1%) had documented metastases. The most frequently observed malignancies were breast cancer (43.4%), and lymphoid or hematopoietic cancers (41.8%). Pre-existing anemia (59.6%), a documented history of infections (43.7%), and pre-existing neutropenia (27.1%) were common. Baseline liver dysfunction was observed in approximately one out of five patients.

### 3.2. Neutropenia-Related Outcomes

Patient distribution and primary outcome data are detailed in Table 2. Among the top 10 ADCs analyzed, the three most frequently administered agents were fam-trastuzumab deruxtecan (20.8%), ado-trastuzumab emtansine (17.8%), and brentuximab vedotin (16.6%). Across all patients, the median number of treatment cycles was 3 (IQR: 7).

The proportion of patients with FN and grade 3 or above neutropenia varied widely by agent. The all-cycle rates of FN were lowest with ado-trastuzumab emtansine (1.2%) and were highest with gemtuzumab ozogamicin (18.1%) and inotuzumab ozogamicin (18.1%). Grade 3+ neutropenia occurred most frequently with gemtuzumab ozogamicin (93.8%), followed by inotuzumab ozogamicin (86.7%), and brentuximab vedotin (40.1%). In contrast, the lowest rates were observed with fam-trastuzumab deruxtecan (16.4%) and ado-trastuzumab emtansine (5.1%).

### 3.3. Healthcare Services Utilization

The use of primary G-CSF prophylaxis varied by ADC, with the highest utilization seen in patients receiving polatuzumab vedotin (34.2%), sacituzumab govitecan (31.2%), and inotuzumab ozogamicin (21.4%) (Table 3).

Hospital and ICU admissions were also most frequent among patients receiving ADCs with higher neutropenia risk, as shown in Table 3. Inotuzumab ozogamicin was associated with the highest rate of hospital admissions (36.7%), ICU admissions (4.3%), and mortality (6.7%). Elevated hospitalization rates were also observed with gemtuzumab ozogamicin (31.3%) and tisotumab vedotin (25.0%). ICU admissions were notable for polatuzumab vedotin (3.1%) and tisotumab vedotin (2.1%). Increased mortality was also documented with polatuzumab vedotin (4.6%) and sacituzumab govitecan (2.2%).

### 3.4. Characteristics Associated with Neutropenia-Related Events

**Figure 1:**
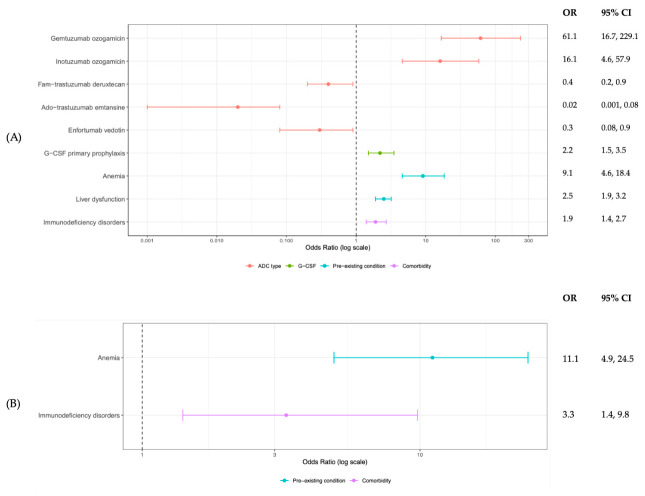
Predictors for First Cycle (**A**) Grade 3 and Above Neutropenia and (**B**) Febrile Neutropenia in All Patients Receiving ADCs.

Fam-trastuzumab deruxtecan, ado-trastuzumab emtansine, and enfortumab vedotin were associated with a lower risk of grade ≥ 3 neutropenia during the first cycle compared with other ADCs. In contrast, patients who received primary prophylaxis with G-CSF during the first cycle were at a higher risk of grade ≥ 3 neutropenia. Additional pre-existing conditions including liver dysfunction and immunodeficiency were also significant predictors of grade ≥ 3 neutropenia. (Figure 1A) Pre-existing anemia prior to ADC initiation was associated with an increased risk of both grade ≥ 3 neutropenia and FN after the first cycle of ADC (Figure 1B).

**Figure 2:**
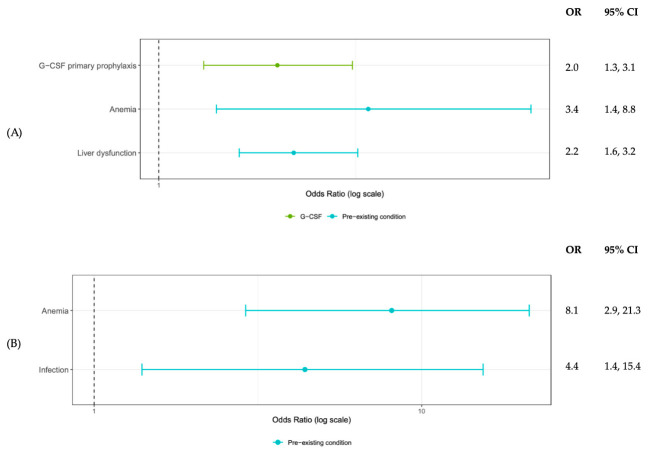
Predictors for First Cycle (**A**) Grade 3 and Above Neutropenia and (**B**) Febrile Neutropenia in Hematological Malignancy Patients Receiving ADCs.

**Figure 3:**
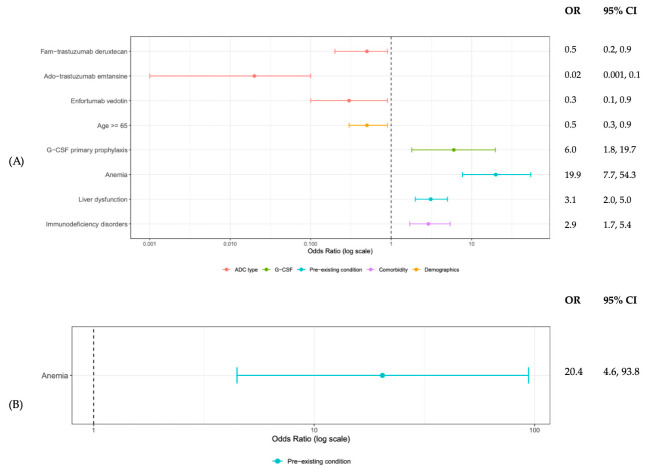
Predictors for First Cycle (**A**) Grade 3 and Above Neutropenia and (**B**) Febrile Neutropenia in Solid Tumor Patients Receiving ADCs.

In stratified analysis of patients with hematological malignancies, primary G-CSF prophylaxis, anemia, and liver dysfunction were associated with a higher risk of first cycle grade ≥ 3 neutropenia. (Figure 2A) Anemia and infection were predicted higher odds for first cycle FN. (Figure 2B) In contrast, the solid tumor model showed a slightly different profile: three ADCs (fam-trastuzumab deruxtecan, ado-trastuzumab emtansine, and enfortumab vedotin) and age ≥ 65 years were associated with a lower risk of grade ≥ 3 neutropenia, whereas G-CSF prophylaxis, anemia, liver dysfunction, and immunodeficiency disorders were associated with an increased risk. (Figure 3A) Anemia was also predictive of a higher risk of first cycle FN among solid tumor patients (Figure 3B).

## 4. Discussion

This retrospective analysis evaluated the safety profiles of the top 10 most commonly used ADCs across six University of California medical centers using real-world data from a 10-year span. Our analysis leverages the University of California Health Data Warehouse, a multi-center electronic health record repository spanning these six academic medical centers. This resource captures a heterogeneous oncology population treated in routine clinical practice, including patients who may not qualify for prospective trials, thereby enhancing the external validity of our findings and reflecting contemporary treatment patterns. Among the 3511 patients included, we observed a higher proportion of neutropenia-related events in patients receiving ADCs for treating hematologic malignancies, likely contributed to by the drugs as well as the underlying cancers [21]. In contrast, ADCs that are utilized for managing solid tumors are linked to lower neutropenia-related events.

Fam-trastuzumab deruxtecan and ado-trastuzumab emtansine were the most prescribed ADCs, and they were also associated with low rates of FN, grade 3 or higher neutropenia, hospital admissions, ICU admissions, and mortality within this cohort. These findings are encouraging and suggest a potentially more manageable toxicity profile of these two ADCs in this real-world population. Their frequent use likely reflects current clinical indications and the higher prevalence of breast cancer in the population studied, rather than inherently lower toxicity [22]. The observed safety outcomes may also be influenced by patient characteristics, earlier lines of therapy, or more established supportive care protocols in solid tumor settings compared to hematologic malignancies. Given the retrospective design, these findings should be interpreted as observations rather than as evidence of comparative safety.

In contrast, gemtuzumab ozogamicin and inotuzumab ozogamicin, which are primarily used in hematologic malignancies, are observed to have high rates of FN and grade 3 or higher neutropenia, along with increased hospitalizations, ICU admissions, and mortality. Gemtuzumab ozogamicin is indicated for acute myeloid leukemia (AML) and acute promyelocytic leukemia (APL), while inotuzumab ozogamicin is indicated for acute lymphoblastic leukemia (ALL) [23,24]. These findings are consistent with published safety data for gemtuzumab ozogamicin, though they are less well established for inotuzumab ozogamicin [11,12,23,24]. The elevated rates of toxicity may reflect not only the myelosuppressive potential of these agents but also the underlying vulnerability of patients with hematologic cancers, who often have a higher baseline risk of neutropenia [25].

Beyond fam-trastuzumab deruxtecan and ado-trastuzumab emtansine, the remaining ADCs in this cohort demonstrated variable safety profiles that reflect observed varying patterns and may be influenced by underlying indications and toxicity patterns. Brentuximab vedotin and polatuzumab vedotin, both used in lymphoid malignancies, are observed to have higher rates of grade 3 or higher neutropenia (40.1% and 40.3%, respectively) and moderate rates of febrile neutropenia. The overall FN rate of brentuximab vedotin is modest at 10.4%, suggesting a need to identify patient-related risk factors to determine whether primary prophylaxis with G-CSF is necessary for specific patients in order to mitigate the neutropenic risk.

Sacituzumab govitecan, an ADC used primarily in bladder cancer and recently approved for breast cancer, had a grade 3 or above neutropenia rate of 28.8% and an FN rate of 3.5%, representing a notable neutropenic burden within this real-world cohort [26]. However, in view of its low FN rate, routine prophylaxis may not be necessary. Enfortumab vedotin, used in urothelial cancer, demonstrated relatively lower rates of febrile and severe neutropenia, though admissions remained notable [27]. Belantamab mafodotin and tisotumab vedotin, which had smaller sample sizes in this analysis, showed modest neutropenia rates but variable inpatient outcomes, warranting further evaluation in larger datasets. These patterns emphasize the importance of considering both disease contexts and ADC-specific toxicity profiles when evaluating risk and tailoring supportive care [21,28,29].

G-CSF prophylaxis utilization patterns further underscore the variability of neutropenic risk among different ADCs. The highest rates of prophylactic use were observed with polatuzumab vedotin (34.2%), sacituzumab govitecan (31.2%), and inotuzumab ozogamicin (21.4%), all of which were also observed in association with clinically significant neutropenic events. These findings reflect the need to improve the strategies to reduce neutropenia-related events in patients receiving these ADCs, including the incorporation of appropriate dose modifications in patients with numerous risk factors [29]. Brentuximab vedotin, though frequently used in clinical practice, is linked to a rather low G-CSF prophylaxis rate at 15.3%, suggesting heterogeneity in supportive care approaches, and room to improve the prescription of G-CSF to reduce neutropenia-related events. Further studies are required to examine whether an increased uptake of G-CSF may help to reduce the neutropenic events that are observed in this study [30].

Numerous factors may explain the varying degrees of hematological toxicities associated with ADCs. Drug-specific factors including the categories and properties of payload, ability to target expressed antigens in the bone marrow, as well as the type of linker of an ADC may contribute to off-target toxicity [29]. Our study observed that patient-specific factors may be associated with clinically significant neutropenia events, with pre-existing anemia being the strongest factor that was observed in all models in prediction of both grade 3 or above neutropenia and FN in our full cohort as well as our stratified models based on malignancy status. This suggests that poor bone marrow reserve consistently contributes to ADC-induced neutropenic events [25].

This study also highlights the need to garner real-world evidence from large integrated health systems to evaluate the hematological adverse events of novel anticancer agents, in order to proactively inform supportive care measures. The use of real-world evidence enables the assessment of adverse events in diverse, unselected patient populations who may be underrepresented in clinical trials. These insights are critical for understanding toxicity patterns in routine oncology care and for supporting more informed treatment decisions. Future studies may include the use of artificial intelligence, such as machine learning methods, to further evaluate factors associated with neutropenic events in patients receiving ADCs to better inform supportive care interventions.

This study has several strengths. The large and diverse patient population drawn from multiple academic centers enhances the generalizability of findings. The inclusion of multiple clinically relevant safety endpoints, including febrile neutropenia, grade 3 or higher neutropenia, prophylactic GCSF use, and inpatient outcomes, provides a comprehensive overview of ADC-related risks in routine clinical practice. Additionally, the focus on ten widely used ADCs allows for comparisons across a broad range of malignancies and treatment contexts. There are a few limitations inherent to the study’s retrospective design and reliance on real-world data from administrative sources. The dataset may contain missing or incomplete clinical information, and causal relationships between ADC exposure and adverse events cannot be established. Important clinical variables such as cancer stage, treatment intent, dosing schedules, performance status, and concomitant therapies are often not available in structured data fields, making it difficult to control for confounding factors that influence neutropenia risk. Furthermore, clinical outcomes such as febrile neutropenia were identified using proxy definitions based on diagnosis codes, laboratory values, and antibiotic use, which may lead to misclassification. In particular, the ICD-based component of our febrile neutropenia definition may introduce bias, as diagnosis codes may not accurately capture clinical severity, the exact timing of events, or variations in provider documentation and coding practices. Lastly, the UC Data Warehouse contains deidentified data and does not include patient identifiers, making validation using the chart review approach not feasible. However, our approach mirrors two published studies that have utilized real-world studies that utilized large administrative and claims databases to evaluate real-world outcomes related to FN and neutropenia-related hospitalizations. Both studies employed claims-based algorithms using diagnosis and treatment codes to operationalize FN in the absence of laboratory or chart-level confirmation [16,17].

## 5. Conclusions

Using real-world data from a large, multi-center database within the University of California system, we observed that neutropenia risks differed substantially across various ADCs. The most prescribed ADCs, fam-trastuzumab deruxtecan and ado-trastuzumab emtansine, were associated with low rates of febrile neutropenia and related complications among all ADCs. However, ADCs predominantly used in hematologic malignancies, including gemtuzumab ozogamicin and inotuzumab ozogamicin, were associated with higher rates of severe neutropenia and downstream complications. With an increased use of ADCs in clinical practice, these findings reinforce the need for heightened monitoring and proactive supportive care strategies, including the consideration of appropriate growth factor support in at-risk populations. Overall, the results highlight the importance of integrating ADC-specific hematologic risk into treatment selection and supportive care planning. Future research should also utilize real-world data to investigate other ADC-related toxicities, such as ocular toxicities, pulmonary toxicities, and neurological toxicities. Continued evaluation using real-world datasets and prospective studies is warranted to refine risk mitigation strategies and optimize outcomes across diverse oncology populations.

## Figures and Tables

**Table 1:** Patient characteristics at baseline.

Characteristic	Value
Age (years): Mean ± SD	56.7 ± 17.6
<65 years	2178 (62.0%)
>65 years	1333 (38.0%)
Sex	
Female	2307 (65.7%)
Male	1204 (34.3%)
Weight	
<60 kg	734 (20.9%)
>60 kg	1964 (55.9%)
Unknown	813 (23.2%)
Race (Top three)	
White	1849 (52.7%)
Asian	503 (14.3%)
Black	182 (5.2%)
Ethnicity	
Hispanic or Latino	767 (21.8%)
Not Hispanic or Latino	2604 (74.2%)
Metastases	2074 (59.1%)
Type of malignancy (Top three)	
Breast	1523 (43.4%)
Lymphoid, hematopoietic	1466 (41.8%)
Urinary tract	471 (13.4%)
Number of comorbidities: Mean ± SD	8.4 ± 3.9
0–3	477 (13.6%)
4–10	1966 (56.0%)
10+	1068 (30.4%)
Type of comorbidities (Top 5)	
GI tract	2788 (79.4%)
Musculoskeletal	2490 (70.9%)
Upper GI Tract	2327 (66.3%)
Endocrine	2094 (59.6%)
Immunodeficiency	2052 (58.4%)
Pre-existing neutropenia	952 (27.1%)
Pre-existing lymphocytopenia	29 (0.8%)
Pre-existing anemia	2093 (59.6%)
Pre-existing infection	1534 (43.7%)
Recent surgery	398 (11.2%)
Baseline liver dysfunction	680 (19.4%)
Baseline renal dysfunction	506 (14.4%)

**Table 2:** Patient distribution and neutropenia-related outcomes.

Antibody-Drug Conjugate	*N* (%)	Febrile Neutropenia		Grade 3+ Neutropenia	
		*n* (%)	95% CI	*n* (%)	95% CI
**ADCs primarily used for solid tumors**					
**Fam-trastuzumab deruxtecan**	795 (22.6%)	43 (5.4%)	4.0–7.2%	130 (16.4%)	13.9–19.1%
**Ado-trastuzumab emtansine**	680 (19.4%)	8 (1.2%)	0.6–2.3%	35 (5.1%)	3.7–7.1%
**Sacituzumab govitecan**	545 (15.5%)	51 (9.4%)	7.2–12.1%	157 (28.8%)	25.2–32.7%
**Enfortumab vedotin**	404 (11.5%)	16 (4.0%)	2.5–6.3%	37 (9.2%)	6.7–12.4%
**Tisotumab vedotin**	48 (1.4%)	1 (2.1%)	0.4–10.9%	4 (8.3%)	3.3–19.6%
**ADCs primarily used for hematologic malignancies**					
**Gemtuzumab ozogamicin**	243 (6.9%)	44 (18.1%)	13.8–23.4%	228 (93.8%)	90.1–96.2%
**Brentuximab vedotin**	634 (18.1%)	66 (10.4%)	8.3–13.0%	254 (40.1%)	36.3–43.9%
**Inotuzumab ozogamicin**	210 (6.0%)	38 (18.1%)	13.5–23.9%	182 (86.7%)	81.4–90.6%
**Polatuzumab vedotin**	196 (5.6%)	12 (6.1%)	3.5–10.4%	79 (40.3%)	33.7–47.3%
**Belantamab mafodotin**	74 (2.1%)	5 (6.8%)	2.9–14.9%	19 (25.7%)	17.1–36.7%

**Table 3:** Rates of healthcare utilization associated with individual ADCs.

Antibody-Drug Conjugate	N (%)	GCSF Prophylaxis		Hospital Admission		ICU Admission		Death	
		n (%)	95% CI	n (%)	95% CI	n (%)	95% CI	n (%)	95% CI
**ADCs primarily used for solid tumors**									
**Fam-trastuzumab deruxtecan**	795 (22.6%)	75 (9.4%)	7.6–11.7%	84 (10.6%)	8.6–12.9%	4 (0.5%)	0.2–1.3%	12 (1.5%)	0.9–2.6%
**Ado-trastuzumab emtansine**	680 (19.4%)	9 (1.3%)	0.7–2.5%	65 (9.6%)	7.6–12.0%	1 (0.1%)	0.0–0.8%	2 (0.3%)	0.1–1.1%
**Sacituzumab govitecan**	545 (15.5%)	170 (31.2%)	27.4–35.2%	93 (17.1%)	14.1–20.5%	5 (0.9%)	0.4–2.1%	12 (2.2%)	1.3–3.8%
**Enfortumab vedotin**	404 (11.5%)	21 (5.2%)	3.4–7.8%	91 (22.5%)	18.7–26.8%	3 (0.7%)	0.3–2.2%	7 (1.7%)	0.8–3.5%
**Tisotumab vedotin**	48 (1.4%)	0 (0.0%)	0.0–7.4%	12 (25.0%)	14.9–38.8%	1 (2.1%)	0.4–10.9%	1 (2.1%)	0.4–10.9%
**ADCs primarily used for hematologic malignancies**									
**Gemtuzumab ozogamicin**	243 (6.9%)	14 (5.8%)	3.5–9.4%	76 (31.3%)	25.8–37.4%	3 (1.2%)	0.4–3.6%	6 (2.5%)	1.1–5.3%
**Brentuximab vedotin**	634 (18.1%)	97 (15.3%)	12.7–18.3%	141 (22.2%)	19.2–25.6%	3 (0.5%)	0.2–1.4%	10 (1.6%)	0.9–2.9%
**Inotuzumab ozogamicin**	210 (6.0%)	45 (21.4%)	16.4–27.5%	77 (36.7%)	30.4–43.4%	9 (4.3%)	2.3–7.9%	14 (6.7%)	4.0–10.9%
**Polatuzumab vedotin**	196 (5.6%)	67 (34.2%)	27.9–41.1%	45 (23.0%)	17.6–29.3%	6 (3.1%)	1.4–6.5%	9 (4.6%)	2.4–8.5%
**Belantamab mafodotin**	74 (2.1%)	3 (4.1%)	1.4–11.3%	12 (16.2%)	9.5–26.2%	1 (1.4%)	0.2–7.3%	1 (1.4%)	0.2–7.3%

## Data Availability

The datasets presented in this article are not readily available because they were obtained from the University of California Health Data Warehouse and are subject to institutional data use agreements and privacy restrictions. Requests to access the datasets should be directed to the UC Health Data Warehouse.

## Supplementary Materials

The following supporting information can be downloaded at: https://www.mdpi.com/article/10.3390/cancers18101563/s1, Table S1: Average time (days, 95% CI) between cancer diagnosis and individual ADC initiation.

## References

[1] Fu Z., Li S., Han S., Shi C., Zhang Y. (2022). Antibody drug conjugate: The “biological missile” for targeted cancer therapy. Signal Transduct. Target. Ther..

[2] Wang R., Hu B., Pan Z., Mo C., Zhao X., Liu G., Hou P., Cui Q., Xu Z., Wang W. (2025). Antibody-Drug Conjugates (ADCs): Current and future biopharmaceuticals. J. Hematol. Oncol..

[3] Corti C., Giugliano F., Nicolò E., Ascione L., Curigliano G. (2021). Antibody-Drug Conjugates for the Treatment of Breast Cancer. Cancers.

[4] Zhang F., Li S. (2025). Antibody-drug conjugates as game changers in bladder cancer: Current progress and future directions. Front. Immunol..

[5] Hassan H.T. (2023). Antibody-drug conjugate [ADC] treatment of leukaemia. Leuk. Res..

[6] Gerber H.P., Gangwar S., Betts A. (2023). Therapeutic index improvement of antibody-drug conjugates. MAbs.

[7] Gogia P., Ashraf H., Bhasin S., Xu Y. (2023). Antibody-Drug Conjugates: A Review of Approved Drugs and Their Clinical Level of Evidence. Cancers.

[8] Zhou K., Liu X., Zhu H. (2025). Overcoming resistance to antibody-drug conjugates: From mechanistic insights to cutting-edge strategies. J. Hematol. Oncol..

[9] Zhu Y., Liu K., Wang K., Zhu H. (2023). Treatment-related adverse events of antibody-drug conjugates in clinical trials: A systematic review and meta-analysis. Cancer.

[10] D’Arienzo A., Verrazzo A., Pagliuca M., Napolitano F., Parola S., Viggiani M., Caputo R., Puglisi F., Giuliano M., Del Mastro L. (2023). Toxicity profile of antibody-drug conjugates in breast cancer: Practical considerations. EClinicalMedicine.

[11] Rao M., Wu L., Chen H., Wu X., Wang H., Chen Y., Chen C. (2025). Hematological toxicity of anti-tumor antibody-drug conjugates: A retrospective pharmacovigilance study using the FDA adverse event reporting system. PLoS ONE.

[12] Wang S., Pan H., Chen Z., Zhou H., Chen J., Zou G., Huang J., Mei Q. (2026). Hematological toxicities in antibody-drug conjugates related with breast cancer: A pharmacovigilance study using FDA adverse event reporting system database. Expert Opin. Drug Saf..

[13] Gui X., Zhao J., Ding L., Chai J., Lai H., Cai Y., Luo S., Zeng Y., Wu W., Chen H. (2023). Assessing real-world safety concerns of Sacituzumab govitecan: A disproportionality analysis using spontaneous reports in the FDA adverse event reporting system. Front. Oncol..

[14] Fountzilas E., Karageorgopoulou S., Karakatsoulis G., Tryfonopoulos D., Papazisis K., Koutras A., Koumarianou A., Zafeiri G., Biziota E., Nikolaidi A. (2025). Real-world safety and effectiveness data of trastuzumab deruxtecan and sacituzumab govitecan in breast cancer: A Hellenic Cooperative Oncology Group study. ESMO Real. World Data Digit. Oncol..

[15] UCLA Clinical and Translational Science Institute UC Health Data Warehouse (UCHDW). Published 15 January 2025. https://ctsi.ucla.edu/uc-health-data-warehouse-uchdw.

[16] Kawatkar A.A., Farias A.J., Chao C., Chen W., Barron R., Vogl F.D., Chandler D.B. (2017). Hospitalizations, outcomes, and management costs of febrile neutropenia in patients from a managed care population. Support Care Cancer.

[17] Family L., Yang S.J., Klippel Z., Li Y., Page J.H., Rodriguez R., Chao C. (2015). Risk of Febrile Neutropenia (FN) in Select Myelosuppressive Chemotherapy Regimens. Blood.

[18] US Department of Health and Human Services (2017). Common Terminology Criteria for Adverse Events (CTCAE) Common Terminology Criteria for Adverse Events (CTCAE) V5.0. https://ctep.cancer.gov/protocoldevelopment/electronic_applications/docs/CTCAE_v5_Quick_Reference_5x7.pdf.

[19] Weycker D., Malin J., Edelsberg J., Glass A., Gokhale M., Oster G. (2008). Cost of neutropenic complications of chemotherapy. Ann. Oncol..

[20] (2025). NCCN Clinical Practice Guidelines in Oncology. Hematopoietic Growth Factors. Version 2.2025 [Internet]. https://www.nccn.org/professionals/physician_gls/pdf/growthfactors.pdf.

[21] Masters J.C., Nickens D.J., Xuan D., Shazer R.L., Amantea M. (2018). Clinical toxicity of antibody-drug conjugates: A meta-analysis of payloads. Investig. New Drugs.

[22] Mukohara T., Hosono A., Mimaki S., Nakayama A., Kusuhara S., Funasaka C., Nakao T., Fukasawa Y., Kondoh C., Harano K. (2021). Effects of Ado-Trastuzumab Emtansine and Fam-Trastuzumab Deruxtecan on Metastatic Breast Cancer Harboring HER2 Amplification and the L755S Mutation. Oncologist.

[23] Larson R.A., Sievers E.L., Stadtmauer E.A., Lowenbery B., Estey E.H., Dombret H., Theobald M., Voliotis D., Bennett J.M., Richie M. (2005). Final report of the efficacy and safety of gemtuzumab ozogamicin (Mylotarg) in patients with CD33-positive acute myeloid leukemia in first recurrence. Cancer.

[24] Kantarjian H.M., DeAngelo D.J., Stelljes M., Martinelli G., Liedtke M., Stock W., Gokbuget N., O’Biren S., Wang K., Wang T. (2016). Inotuzumab Ozogamicin versus Standard Therapy for Acute Lymphoblastic Leukemia. N. Engl. J. Med..

[25] Lakshmaiah K.C., Malabagi A.S., Govindbabu Shetty R., Sinha M., Jayashree R.S. (2015). Febrile Neutropenia in Hematological Malignancies: Clinical and Microbiological Profile and Outcome in High Risk Patients. J. Lab. Physicians.

[26] Pérez-García J.M., Gion M., Ruiz-Borrego M., Blancas I., López-Miranda E., Blanch S., Recalde S., Rendo C.R., González X., Ancizar N. (2025). Prevention of sacituzumab govitecan-related neutropenia and diarrhea in patients with HER2-negative advanced breast cancer (PRIMED): An open-label, single-arm, phase 2 trial. EClinicalMedicine.

[27] Otsuka A., Sawada N., Suda R., Yano F., Osada T., Otake Y., Shimura H., Mochizuki T., Harada D., Goto J. (2024). Enfortumab Vedotin-Induced Febrile Neutropenia and Hyperglycemia Successfully Treated with Multidisciplinary Treatment Including Continuous Hemodialysis Filtration and Insulin Injection in a Patient with Chemo-Resistant Metastatic Urothelial Carcinoma: A Case Report. Case Rep. Oncol..

[28] Cheng Y., Lu J., Zhang C., Yan W., Zhu P., Qin Q., Gong L. (2025). Overview of antibody-drug conjugates nonclinical and clinical toxicities and related contributing factors. Antib. Ther..

[29] Suzuki Y., Zhou S., Ota Y., Harrington M., Miyagi E., Takagi H., Kuno T., Wright J.D. (2023). Toxicity profiles of antibody-drug conjugates for anticancer treatment: A systematic review and meta-analysis. JNCI Cancer Spectr..

[30] Straus D.J., Długosz-Danecka M., Connors J.M., Alekseev S., Illés Á., Picardi M., Lech-Maranda E., Feldman T., Smolewski P., Savage K.J. (2020). Primary prophylaxis with G-CSF may improve outcomes in patients with newly diagnosed advanced-stage Hodgkin lymphoma treated with brentuximab vedotin plus chemotherapy. Br. J. Haematol..

