# Supplementary material for: Incidence of Clinically Significant Neutropenia and Complications Related to Antibody-Drug Conjugates: A Real-World Study at the University of California
Source: Cancers (Basel). 2026 May 12;18(10):1563. doi: 10.3390/cancers18101563 (PMC13204587; doi:10.3390/cancers18101563)
Supplement: Supplementary file 1 [file cancers-18-01563-s001.zip › cancers-4263776-supplementary.pdf]

Table S1: Average time (days, 95% CI) between cancer diagnosis and individual ADC initiation

Antibody Drug Conjugate	Average Time to Initiation (Days, 95% CI)
Fam-trastuzumab deruxtecan	1440 (1375, 1504)
Ado-trastuzumab emtansine	644 (590,699)
Brentuximab vedotin	469 (410, 528)
Sacituzumab govitecan	1328 (1248, 1407)
Enfortumab vedotin	892 (811, 972)
Gemtuzumab ozogamicin	290 (218, 363)
Inotuzumab ozogamicin	605 (513, 697)
Polatuzumab vedotin	733 (624, 843)
Belantamab mafodotin	1655 (1428, 1883)
Tisotumab vedotin	1052 (868, 1236)
